# Ectopic Expression of O Antigen in *Bordetella pertussis* by a Novel Genomic Integration System

**DOI:** 10.1128/mSphere.00417-17

**Published:** 2018-01-24

**Authors:** Keisuke Ishigaki, Naoaki Shinzawa, Sayaka Nishikawa, Koichiro Suzuki, Aya Fukui-Miyazaki, Yasuhiko Horiguchi

**Affiliations:** a Department of Molecular Bacteriology, Research Institute for Microbial Diseases, Osaka University, Osaka, Japan; b Research Foundation for Microbial Diseases of Osaka University, Osaka, Japan; University of Oslo

**Keywords:** *Bordetella*, *Bordetella bronchiseptica*, *Bordetella pertussis*, O antigen, site-specific recombination

## Abstract

We describe a novel genome integration system that enables the introduction of DNA fragments as large as 50 kbp into the chromosomes of recipient bacteria. This system, named BPI, comprises a bacterial artificial chromosome vector and phage-derived gene integration machinery. We introduced the *wbm* locus of *Bordetella bronchiseptica*, which is required for O antigen biosynthesis, into the chromosome of *B. pertussis*, which intrinsically lacks O antigen, using the BPI system. After the introduction of the *wbm* locus, *B. pertussis* presented an additional substance in the lipooligosaccharide fraction that was specifically recognized by the anti-*B. bronchiseptica* antibody but not the anti-*B. pertussis* antibody, indicating that *B. pertussis* expressed O antigen corresponding to that of *B. bronchiseptica*. O antigen-expressing *B. pertussis* was less sensitive to the bactericidal effects of serum and polymyxin B than the isogenic parental strain. In addition, an *in vivo* competitive infection assay showed that O antigen-expressing *B. pertussis* dominantly colonized the mouse respiratory tract over the parental strain. These results indicate that the BPI system provides a means to alter the phenotypes of bacteria by introducing large exogenous DNA fragments.

**IMPORTANCE** Some bacterial phenotypes emerge through the cooperative functions of a number of genes residing within a large genetic locus. To transfer the phenotype of one bacterium to another, a means to introduce the large genetic locus into the recipient bacterium is needed. Therefore, we developed a novel system by combining the advantages of a bacterial artificial chromosome vector and phage-derived gene integration machinery. In this study, we succeeded for the first time in introducing a gene locus involved in O antigen biosynthesis of *Bordetella bronchiseptica* into the chromosome of *B. pertussis*, which intrinsically lacks O antigen, and using this system we analyzed phenotypic alterations in the resultant mutant strain of *B. pertussis*. The present results demonstrate that this system successfully accomplished the above-described purpose. We consider this system to be applicable to a number of bacteria other than *Bordetella*.

## Abstract (precis)

Some bacterial phenotypes emerge through the cooperative functions of a number of genes residing within a large genetic locus. To transfer the phenotype of one bacterium to another, a means to introduce the large genetic locus into the recipient bacterium is needed. Therefore, we developed a novel system by combining the advantages of a bacterial artificial chromosome vector and phage-derived gene integration machinery. In this study, we succeeded for the first time in introducing a gene locus involved in O antigen biosynthesis of *Bordetella bronchiseptica* into the chromosome of *B. pertussis*, which intrinsically lacks O antigen, and using this system we analyzed phenotypic alterations in the resultant mutant strain of *B. pertussis*. The present results demonstrate that this system successfully accomplished the above-described purpose. We consider this system to be applicable to a number of bacteria other than *Bordetella*.

## INTRODUCTION

*Bordetella pertussis*, *Bordetella parapertussis*, and *Bordetella bronchiseptica*, which are the “classical” bordetellae, cause respiratory diseases in mammals (1). Although they are closely related pathogens, their host ranges completely differ. *B. pertussis* is a strictly human-adapted pathogen that causes whooping cough. *B. parapertussis* is composed of two distinct lineages: one lineage is human adapted and causes whooping cough-like disease, while the other lineage only infects sheep (2). *B. bronchiseptica* infects a broad range of mammals and causes various diseases, such as kennel cough in dogs, snuffles in rabbits, and atrophic rhinitis in pigs (1, 3). The underlying pathogenic mechanisms for the different host specificities among *Bordetella* species, which share many virulence factors with each other, have yet to be elucidated.

Recent comparative genome analyses on closely related pathogens revealed a genetic background that involves the adaptation of pathogens to the host environment. In *Yersinia pestis*, the acquisition of the plasmid pPCP1 containing the gene *pla*, which encodes a protease, is an important step for adaptation to the pulmonary environment in host animals (4). A loss-of-function mutation in a gene for urease also provides a new niche for *Y. pestis* (5). The host tropism of *Staphylococcus aureus* and *Salmonella enterica* serovar Typhimurium are reportedly influenced by a single mutation in a gene that encodes a membrane protein and an allelic variation in an adhesin, respectively (6, 7).

In the case of *Bordetella*, previous studies indicated that *B. pertussis* and *B. parapertussis* independently evolved from *B. bronchiseptica*-like ancestors through large-scale gene losses and translocations and, as a result, the genome sizes of *B. pertussis* and *B. parapertussis* became approximately 1.3 Mbp and 0.6 Mbp smaller, respectively, than that of *B. bronchiseptica* (8–10). Limited evidence is currently available for the large acquisition of genes through horizontal gene transfer during evolution (8–10). Differences in host tropism among *Bordetella* species have been suggested to result from gene decay. If this is the case, the narrow host specificity of *B. pertussis* may be altered by supplying a number of genes of *B. bronchiseptica*, which has a wide range of host animals. We decided to examine this possibility. To achieve this, we needed an efficient means to enable the introduction of the large genomic DNA (gDNA) fragments of *B. bronchiseptica* into the chromosome of *B. pertussis*.

In the present study, we propose a novel method to achieve this purpose; we named it the BPI system. This method is based on the combination of a bacterial artificial chromosome (BAC) vector and phage-derived integration machinery. To verify the usability of the BPI system, we attempted to generate O antigen-expressing *B. pertussis*, the parental strain of which does not intrinsically express O antigen, by introducing the *B. bronchiseptica wbm* gene locus, which is approximately 32 kbp (11–13). The resultant *B. pertussis* strain expressed the O antigen of *B. bronchiseptica* and became more resistant to complement- or polymyxin B-mediated bactericidal effects than the parental *B. pertussis*. In addition, an *in vivo* competitive infection assay showed that the expression of O antigen contributed to the efficient colonization of *B. pertussis* in the mouse respiratory tract. These results demonstrated that the BPI system has potential as a powerful tool to transform phenotypes through the introduction of large DNA fragments into the chromosomes of recipient bacteria.

## RESULTS

### Construction of the BPI system in *Bordetella* species.

**FIG 1:**
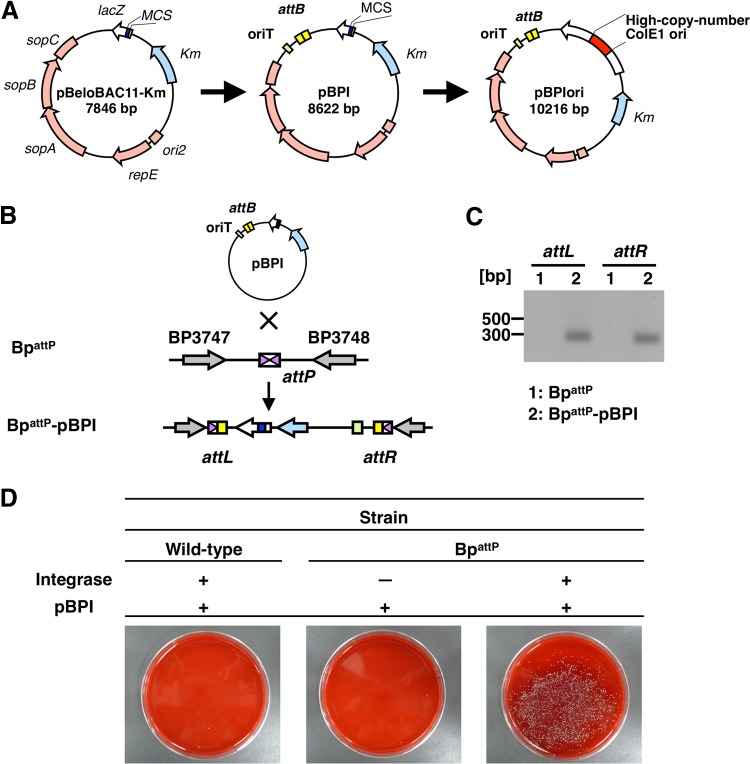
Construction of the BPI system for large genomic DNA integration. (A) Construction of pBPI. pBeloBAC11 was modified for the BPI system. oriT for conjugative transfer and *attB* for site-specific integration were introduced into pBeloBAC11-Km. High-copy-number-yielding ColE1 ori was cloned into the BamHI site of pBPI in order to obtain a large amount of the plasmid (pBPIori). The ColE1 ori was removed by subsequent BamHI treatment, and the resulting plasmid was identical to pBPI. (B) Schematic diagram of the BPI system. pBPI was integrated into the chromosome of Bp^attP^ via* attB* on pBPI and *attP* on the chromosome by an ectopically expressed integrase. *attL* and *attR* regions are junction regions generated after integration. (C) The integration of pBPI into the Bp^attP^ chromosome was verified with PCR. PCR was performed using the genomes of Bp^attP^ and Bp^attP^-pBPI as the templates. As judged by electrophoresis, the newly generated *attL* and *attR* sites after integration were only amplified when the latter template was used. (D) Colonies of *B. pertussis* grown on agar plates containing kanamycin. Only Bp^attP^ harboring the integrase-expressing plasmid (pBBR1MCS5-Int) grew after the introduction of pBPI.

We constructed the vector for the BPI system by combining the advantages of the BAC vector to carry large DNA fragments (14) and the phiC31 phage to integrate these fragments into a bacterial chromosome in a site-specific manner (15) (Fig. 1A and B). We initially introduced a kanamycin (Km) resistance gene into pBeloBAC11 in place of the chloramphenicol resistance gene, because resistance to chloramphenicol is often unreproducible in *Bordetella* grown on Bordet-Gengou (BG) agar plates. The resultant plasmid was designated pBeloBAC11-Km. An origin of transfer (oriT), which is necessary for the conjugative transfer of the vector from *Escherichia coli* to *B. pertussis*, was subsequently introduced. To utilize the integration machinery of the phiC31 phage, we also placed the attachment sites of the phage (*attP*) and bacterium (*attB*) into the chromosome of *B. pertussis* and pBeloBAC11-Km, respectively. The strain and plasmid obtained were designated Bp^attP^ and pBPI. The ColE1 origin of replication (ori) was also introduced into pBPI from high-copy-number pBlueScript to obtain the plasmid from *E. coli* at a high yield. The resulting plasmid was designated pBPIori. The ColE1 ori was removed from pBPIori via a treatment with BamHI before use. The plasmid after the removal of the ColE1 ori was expected to be identical to pBPI.

We initially attempted to introduce pBPI itself into Bp^attP^ carrying pBBR1MCS5-Int, which expresses the phiC31 integrase, to test whether this system functions properly in *B. pertussis*. After the introduction of pBPI, we successfully isolated the Km-resistant colonies of Bp^attP^ (Bp^attP^-pBPI) only when the integrase-expressing plasmid was introduced (Fig. 1D). PCR with primers specific to the junction sequences *attL* and *attR*, which are generated after integration, revealed integration that was mediated by *attB* and *attP* (Fig. 1C). Therefore, the BPI system appeared to function as expected.

### Introduction of large *B. bronchiseptica* gDNAs into the *B. pertussis* chromosome.

The BAC system is capable of maintaining high-molecular-weight DNA in *E. coli* (14). To examine the cloning capacity of pBPI, we ligated large DNA fragments of *B. bronchiseptica* with pBPI and introduced plasmids into *E. coli*. We recovered the plasmids from 21 Km-resistant clones that were randomly picked, we roughly evaluated the sizes of the inserted fragments from the results of electrophoresis after BamHI digestion, and we found that the inserted fragments ranged between 8 and 50 kbp. Sequencing of the largest fragment revealed that it covered an approximately 49.6-kbp region, including the genes BB3602 to BB3665. The plasmid carrying this fragment was named pBPI-L1.

**FIG 2:**
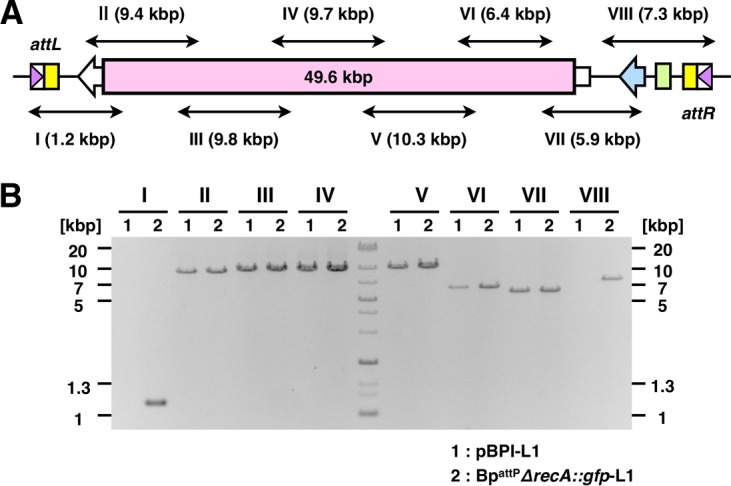
Introduction of a large genomic DNA fragment into the *B. pertussis* chromosome. (A) Schematic diagram of the 49.6-kbp fragment of *B. bronchiseptica* gDNA integrated into the *B. pertussis* chromosome. *attL* and *attR* were junction regions generated after integration. Double-headed arrows (I to VIII) indicate the target regions that were expected to be amplified by PCR. The sizes of the regions are shown in parentheses. (B) PCR amplification of target fragments. Note that fragments I and VIII, flanking the 49.6-kbp fragment, were amplified when the chromosome of Bp^attP^*ΔrecA*::*gfp* after the transformation of pBPI-L1 (Bp^attP^*ΔrecA*::*gfp*-L1), but not pBPI-L1 itself, was used as the template.

Following the conjugative transfer of pBPI-L1 into Bp^attP^, we examined the integration of the vector via PCR by using pBPI-L1-integrated *B. pertussis* gDNA as a template. Although the *attL* and *attR* regions were amplified, the *B. bronchiseptica* gDNA region was not (data not shown), indicating undesirable homologous recombination, possibly because of large homologous sequences between the 49.6-kbp fragment and the chromosome of *B. pertussis*. To overcome this issue, we generated a *recA* deletion mutant of Bp^attP^ (Bp^attP^*ΔrecA*::*gfp*), because RecA is mainly involved in recombination in bacteria (16, 17). Bp^attP^*ΔrecA*::*gfp* did not show any growth defect during *in vitro* culture (see Fig. S1 in the supplemental material). We then transferred pBPI-L1 into Bp^attP^*ΔrecA*::*gfp* in the presence of pBBR1MCS5-Int and checked the integration of pBPI-L1 by PCR performed with pBPI-L1 itself or with the gDNA of Bp^attP^*ΔrecA*::*gfp* after transformation as the template. In this PCR, we used pairs of primers to amplify short fragments, named I to VIII. Fragments II to VII, covering the region of *B. bronchiseptica* gDNA, were amplified by PCR with each template (Fig. 2A and B). In contrast, fragments I and VIII, containing *attL* and *attR*, were only amplified when the gDNA of Bp^attP^*ΔrecA*::*gfp* after transformation (Bp^attP^*ΔrecA*::*gfp*-L1) was used as the template. These results indicated that full-length pBPI-L1 containing the 49.6-kbp fragment of *B. bronchiseptica* gDNA was successfully integrated into the chromosome of *B. pertussis*. Thus, the BPI system is expected to be capable of integrating gDNA fragments as large as 50 kbp into the chromosome of *B. pertussis*.

10.1128/mSphere.00417-17.2 FIG S1  Growth curves of *B. pertussis* wild-type and mutant strains. Bacterial strains were grown at 37°C in Stainer-Scholte medium. Each bar represents the mean ± standard deviation from three individual samples. Some bars are hidden by plot symbols because of small values for the standard deviation. Data were statistically analyzed by the paired Student *t* test, and no significant differences were noted (*P* > 0.05). Download FIG S1, TIF file, 1.1 MB. Copyright © 2018 Ishigaki et al. 2018 Ishigaki et al. This content is distributed under the terms of the Creative Commons Attribution 4.0 International license.

### Introduction of the *wbm* locus of *B. bronchiseptica* into *B. pertussis* via the BPI system.

**FIG 3:**
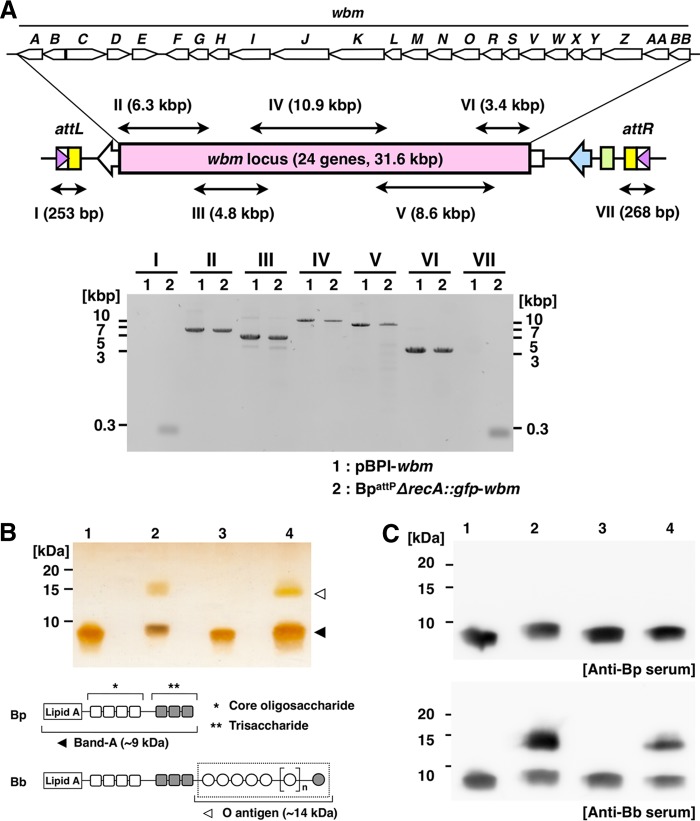
The BPI system complemented O antigen to *B. pertussis*. (A, upper panel) Schematic diagram of the *B. bronchiseptica wbm* locus inserted into the chromosome of *B. pertussis* (12). Double-headed arrows (I to VII) indicate the target regions that were expected to be amplified by PCR. The sizes of the regions are shown in parentheses. (Lower panel) PCR amplification of target fragments. Note that fragments I and VII, including the *attL* and *attR* sites, respectively, were amplified when the chromosome of Bp^attP^*ΔrecA*::*gfp* after the transformation of pBPI-*wbm* (Bp^attP^*ΔrecA*::*gfp*-pBPI), but not pBPI-*wbm* itself, was used as the template. (B, upper panel) Tricine-SDS–PAGE of LPS preparations. LPS components were visualized by silver staining after electrophoresis. Lane 1, *B. pertussis* wild type; lane 2, *B. bronchiseptica* wild type; lane 3, Bp^attP^*ΔrecA*::*gfp*-pBPI; lane 4, Bp^attP^*ΔrecA*::*gfp*-*wbm*. (Lower panel) Schematic representation of the LPS structure of *Bordetella*. The diagram shows an abbreviated arrangement of LPS without the branched saccharide chain (11, 19–21). The region surrounded by the dotted line represents O antigen in *B. bronchiseptica*, and it corresponds to the upper band shown in panels B and C. *B. bronchiseptica* and *B. pertussis* possess similar band A structures, which consist of lipid A, a core oligosaccharide, and trisaccharide. This corresponds to the lower band shown in panels B and C. (C) Western blot analysis of LPS preparations. After electrophoresis and electrotransfer, membranes were incubated with rabbit antiserum against *B. bronchiseptica* and *B. pertussis* diluted 3,000- and 5,000-fold, respectively. Lane 1, *B. pertussis* wild type; lane 2, *B. bronchiseptica* wild type; lane 3, Bp^attP^*ΔrecA*::*gfp*-pBPI; lane 4, Bp^attP^*ΔrecA*::*gfp*-*wbm*.

To assess the usability of the BPI system, we attempted to introduce a functional genetic locus into the chromosome of *B. pertussis*. We selected the *wbm* locus, which comprises 24 genes and has a length of approximately 32 kbp in *B. bronchiseptica* RB50 (12, 18) and is one of the gene clusters lost during evolution of *B. pertussis* (10, 13). The *wbm* locus is also known to be involved in the biosynthesis of O antigen, which is the outermost component of lipopolysaccharide (LPS). *B. pertussis* does not have O antigen because of the lack of the *wbm* locus. Therefore, we attempted to introduce the *wbm* locus into the chromosome of *B. pertussis* by using the BPI system (Fig. 3A). If the genes inserted by the BPI system are functional, O antigen should be expressed in *B. pertussis* recipients. We constructed pBPI carrying the *wbm* locus (pBPI-*wbm*) and introduced it into Bp^attP^*ΔrecA*::*gfp*. The incorporation of pBPI-*wbm* into the chromosome was verified (Fig. 3A) in a series of PCRs in a manner similar to those shown in Fig. 2, and LPS of the tested strains was examined for the expression of O antigen by using Tricine-SDS–PAGE followed by silver staining. *B. bronchiseptica* and *B. pertussis* share similar lipid A and core structures, called band A (19–21) (Fig. 3B, lower panel). While all of the tested strains showed the band A unit at the position of approximately 9 kDa, as reported previously (20, 21), O antigens of approximately 14 kDa were detected in *B. bronchiseptica* wild-type and *wbm*-integrated *B. pertussis* (Bp^attP^*ΔrecA*::*gfp*-*wbm*) strains, but not in the *B. pertussis* wild-type or pBPI-integrated *B. pertussis* (Bp^attP^*ΔrecA*::*gfp*-pBPI) strain (Fig. 3B, upper panel). In addition, Western blot analysis revealed that the 14-kDa band in LPS extracted from Bp^attP^*ΔrecA*::*gfp*-*wbm* was recognized by anti-*B. bronchiseptica* serum but not by anti-*B. pertussis* serum (Fig. 3C), suggesting that Bp^attP^*ΔrecA*::*gfp*-*wbm* expressed O antigen corresponding to that of *B. bronchiseptica*.

### Characterization of O antigen-expressing *B. pertussis*.

**FIG 4:**
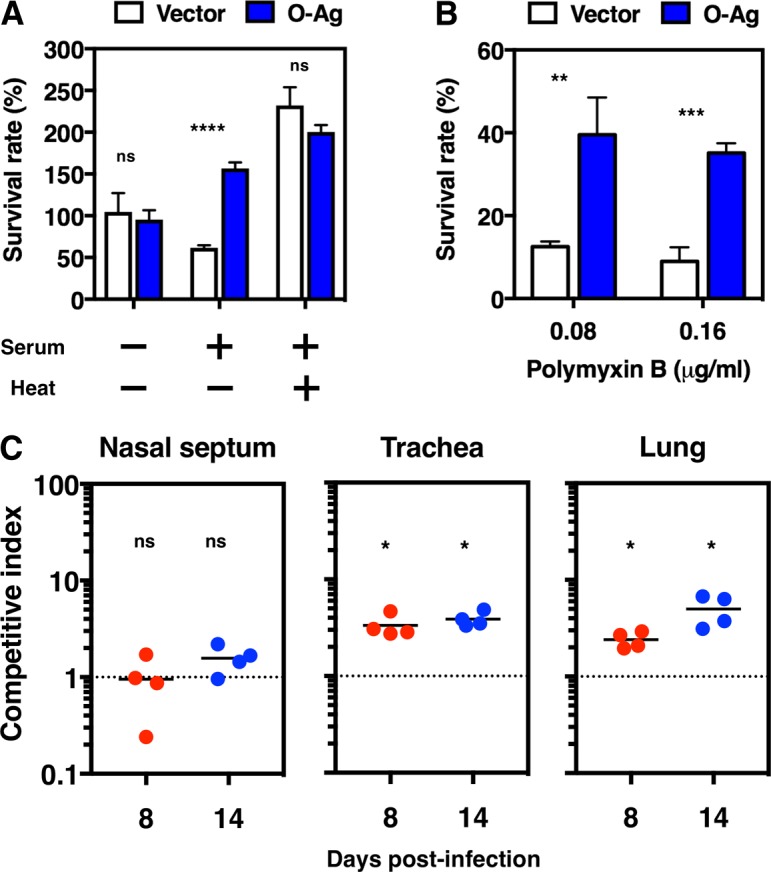
Expression of O antigen conferred resistance against serum and the antimicrobial agent polymyxin B *in vitro* and a fitness advantage in the mouse infection model. (A) Bp^attP^*ΔrecA*::*gfp*-pBPI (vector) and Bp^attP^*ΔrecA*::*gfp*-*wbm* (O-Ag) were incubated at 37°C for 6 h in the presence or absence of 90% intact or heat-inactivated rat serum. Survival rates were measured as described in Materials and Methods. Representative data from three independent experiments are shown. Each bar represents the mean ± standard deviation of three individual samples. Data were statistically analyzed by the paired Student *t* test. ****, *P* < 0.0001; ns, not significant (*P* > 0.05). (B) Bp^attP^*ΔrecA*::*gfp*-pBPI (vector) and Bp^attP^*ΔrecA*::*gfp*-*wbm* (O-Ag) were incubated with polymyxin B at the indicated concentrations at 37°C for 2 h as described in Materials and Methods. Survival rates after the polymyxin B treatment were calculated relative to number of bacteria incubated without polymyxin B (taken as 100%). Representative data from three independent experiments are shown. Each bar represents the mean ± standard deviation of three individual samples. Data were statistically analyzed by the paired Student *t* test. **, *P* < 0.01; ***, *P* < 0.001. (C) CI values for infection of the nasal septum, trachea, and lungs. Groups of four mice were intranasally inoculated with an equally mixed bacterial suspension of Bp^attP^*ΔrecA*::*gfp*-pBPI-Gm and Bp^attP^*ΔrecA*::*gfp*-*wbm*. The organs were excised 8 or 14 days postinfection. CI values were calculated as described in Materials and Methods. Each symbol shows the CI value obtained for an individual mouse. The dashed line indicates the reference CI of 1.00. A CI of >1 indicates that Bp^attP^*ΔrecA*::*gfp*-*wbm* outcompeted Bp^attP^*ΔrecA*::*gfp*-pBPI-Gm. Data were statistically analyzed by a nonparametric Mann-Whitney U test, with the means compared to the hypothetical CI of 1. *, *P* < 0.05; ns, not significant (*P* > 0.05).

Previous studies suggested that O antigen plays a role in the protection of Gram-negative bacteria from host immune responses, such as complement-mediated killing (22–24). Therefore, we examined whether the expressed O antigen conferred some protective ability on *B. pertussis* against serum killing. Approximately 40% of Bp^attP^*ΔrecA*::*gfp*-pBPI was killed by treatment with 90% rat serum, whereas Bp^attP^*ΔrecA*::*gfp*-*wbm* not only survived but also multiplied, even in the presence of serum (Fig. 4A). These two *B. pertussis* derivatives grew similarly in the presence of 90% heat-inactivated serum (Fig. 4A). Polymyxin B, a cationic antimicrobial peptide, interacts with the lipid A moiety of LPS and exhibits potent antibacterial activity (25, 26). We also found that approximately 40% of Bp^attP^*ΔrecA*::*gfp*-*wbm* cells survived in the presence of 0.08 or 0.16 μg/ml of polymyxin B, whereas approximately 10% of Bp^attP^*ΔrecA*::*gfp*-pBPI cells survived under the same conditions (Fig. 4B).

### Contribution of O antigen to respiratory infections in mice.

Since O antigen-expressing *B. pertussis* was more resistant to the bactericidal activity of serum than vector-integrated *B. pertussis*, the former may colonize host animals more efficiently than the latter. To examine this possibility, we performed a competitive infection assay in mice. After the inoculation of equal amounts of Bp^attP^*ΔrecA*::*gfp*-pBPI-Gm and Bp^attP^*ΔrecA*::*gfp*-*wbm*, competitive index (CI) values were calculated from CFU isolated from the nasal septum, trachea, and lungs 8 and 14 days postinfection. Bp^attP^*ΔrecA*::*gfp*-pBPI-Gm and Bp^attP^*ΔrecA*::*gfp*-*wbm* equally colonized the nasal cavity, as judged by CI values of ~1 on days 8 and 14 (Fig. 4C). In contrast, Bp^attP^*ΔrecA*::*gfp*-*wbm* outcompeted Bp^attP^*ΔrecA*::*gfp*-pBPI-Gm in the trachea and lungs, showing mean CI values of 3.33 (day 8) and 3.88 (day 14) and of 2.41 (day 8) and 4.99 (day 14), respectively (Fig. 4C). Bp^attP^*ΔrecA*::*gfp*-pBPI and Bp^attP^*ΔrecA*::*gfp*-pBPI-Gm, which carry kanamycin and gentamicin resistance genes, respectively, in their genomes, were subjected to a similar competitive assay to assess the influence of drug resistance genes on the infectivities of the bacteria, and CI values of ~1 on days 8 and 14 were obtained, indicating that these genes do not affect the ability of these bacteria to colonize mouse respiratory organs (Fig. S2). Collectively, these results indicated that the ability of O antigen-expressing *B. pertussis* to colonize the lower respiratory tract was superior to that of the parental strain.

10.1128/mSphere.00417-17.3 FIG S2  Competitive indices (CI) for infection of the nasal septum, trachea, and lungs. Groups of three mice were intranasally inoculated with an equally mixed bacterial suspension of Bp^attP^*ΔrecA*::*gfp*-pBPI and Bp^attP^*ΔrecA*::*gfp*-pBPI-Gm. The organs were excised 8 or 14 days postinfection. CI values were calculated as described in Materials and Methods in the main text. Each symbol shows the CI value obtained for an individual mouse. The dashed line indicates the reference CI of 1.00. A CI of >1 indicates that Bp^attP^*ΔrecA*::*gfp*-pBPI outcompeted Bp^attP^*ΔrecA*::*gfp*-pBPI-Gm. Data were statistically analyzed using a nonparametric Mann-Whitney U test, with the means compared to the hypothetical CI of 1. ns, not significant (*P* > 0.05). Download FIG S2, TIF file, 1.1 MB. Copyright © 2018 Ishigaki et al. 2018 Ishigaki et al. This content is distributed under the terms of the Creative Commons Attribution 4.0 International license.

## DISCUSSION

In the present study, we developed a novel genome integration system, named the BPI system. To accomplish this, we incorporated the features of various genes related to DNA maintenance and transfer, as follows.

We utilized phiC31 integrase, which mediates unidirectional and site-specific recombination between the attachment sites *attP* and *attB* (15). Since no host factor is required for this reaction, this recombination system is applicable to genetic engineering in bacteria and human cells (27–29). In the present study, we constructed the novel cloning vector pBPI by introducing *attB* into pBeloBAC11-Km and *attP* into the chromosome of *B. pertussis* (Fig. 1A and B), based on a previous study showing that the integration efficiency of *attB*-containing DNA into an *attP*-containing chromosome was higher than that for *attP*-containing DNA integration into an *attB*-containing chromosome (30).

BAC vectors, including pBeloBAC11, are derived from the *E. coli* F factor and are maintained at a single or few copies in an *E. coli* cell. This feature makes BACs useful for the cloning and stable maintenance of large DNA fragments in *E. coli*, and they are widely used for the construction of various genomic libraries (31–34). On the other hand, low-copy-number plasmids are difficult to obtain in large numbers and are unsuitable for subsequent cloning procedures (35). To overcome this disadvantage, we introduced the ColE1 ori of high-copy-number pBlueScript into the BamHI site of pBPI (Fig. 1A). The final yield of the resultant plasmid, named pBPIori, was approximately 50-fold higher than that of pBPI. Since the whole region of ColE1 ori is easily removed by BamHI digestion and is not carried over into subsequent cloning procedures, we were able to use pBPIori without adverse effects on vector preparation.

In the first attempt to apply the BPI system, we succeeded in introducing pBPI into the chromosome of *B. pertussis*, as shown in Fig. 1C and D. However, when pBPI carrying the gDNA fragment of *B. bronchiseptica* was used, genomic integration was unsuccessful (data not shown), suggesting unexpected recombination between homologous regions of the chromosome of *B. pertussis* and the gDNA fragment of *B. bronchiseptica* (8–10). To prevent this unwanted recombination, we generated a *recA* deletion mutant of *B. pertussis*. RecA is known to mediate DNA recombination between two homologous regions (16, 17, 36). By using the *recA* deletion mutant, we successfully introduced an approximately 50-kbp fragment of *B. bronchiseptica* DNA into the chromosome of *B. pertussis* (Fig. 2A and B).

To examine whether the BPI system is of practical use, we attempted to introduce the *wbm* locus of *B. bronchiseptica* into *B. pertussis*. This locus is involved in the biosynthesis of the O antigen of LPS and is 32 kbp, a size that is applicable to the BPI system (12). LPS is one of the major components of the outer membrane of Gram-negative bacteria and generally consists of three representative components: lipid A, a core oligosaccharide, and O antigen (37). Although *B. bronchiseptica* and *B. pertussis* share the basic structure of lipid A, a core oligosaccharide, and trisaccharide, the latter does not express O antigen due to the loss of the entire *wbm* locus (12, 18). After the introduction of the *wbm* locus into the chromosome via the BPI system, *B. pertussis* expressed O antigen corresponding to that of *B. bronchiseptica* (Fig. 3B and C). These results indicated that the BPI system introduces a large DNA fragment into the chromosome of the recipient bacterium without genetic damage.

The ectopic expression of O antigen conferred resistance to serum and polymyxin B on *B. pertussis* (Fig. 4A and B). Complement activation is known to occur on the surface of pathogens, and polymyxin B, which is a cationic antimicrobial peptide, exhibits bactericidal activity through interactions with the negatively charged lipid A moiety of LPS (26). Therefore, our results suggest that the expressed O antigen reduces the accessibility of complement and polymyxin B; however, further studies are needed to clarify the mechanisms underlying the protective role of O antigen. O antigen-expressing *B. pertussis* colonized the mouse trachea and lungs better than the isogenic parental strain, suggesting that the expression of O antigen is specifically advantageous for *B. pertussis* during lower respiratory tract infections in mice. On the other hand, the amount of the O antigen-expressing strain recovered from the nasal septum of mice was similar to that of the isogenic parental strain, indicating that the presence of O antigen has a negligible impact on the ability of bordetellae to colonize the nasal cavity. These results are consistent with previous studies in which *wbm*-deleted mutants of *B. bronchiseptica* and *B. parapertussis* were characterized (22). In the present study, we succeeded, for the first time, in characterizing O antigen-expressing *B. pertussis* by using the BPI system.

Collectively, the present results indicate that the BPI system, which alters the phenotype of *B. pertussis* by introducing the large *wbm* locus to generate O antigen, provides us with a means to generate a genomic library carrying the large gDNA fragments of *B. bronchiseptica* into *B. pertussis*. Using the BPI system, we are currently attempting to screen the genetic loci that influence the host tropism of *Bordetella* species. 

## MATERIALS AND METHODS

### Bacterial strains and plasmids.

All bacterial strains and plasmids used in this study are shown in Table S1 and S2. *B. bronchiseptica* RB50 was provided by Peggy A. Cotter (University of North Carolina), and *B. pertussis* Tohama I was maintained in our laboratory. *B. pertussis* and *B. bronchiseptica* strains were grown at 37°C in Stainer-Scholte (SS) broth or BG agar (Becton, Dickinson) containing 1% glycerol and 15% defibrinated horse blood. *E. coli* strains were grown at 37°C in Luria-Bertani (LB) broth or LB agar. Gentamicin, kanamycin, and ceftibuten were added at a final concentration of 10 μg/ml when necessary. The bacterial cell numbers of *B. pertussis* were estimated based on optical density values at 650 nm (OD_650_) of fresh cultures, according to the following equation: OD_650 _of 1.0 = 3.3 × 10^9^ cells/ml. The experimental procedures for construction of mutant strains and plasmids are described in Text S1.

10.1128/mSphere.00417-17.4 TABLE S1  Strains and plasmids used in this study. Download TABLE S1, DOCX file, 0.04 MB. Copyright © 2018 Ishigaki et al. 2018 Ishigaki et al. This content is distributed under the terms of the Creative Commons Attribution 4.0 International license.

10.1128/mSphere.00417-17.5 TABLE S2  Primers used in this study. Download TABLE S2, DOCX file, 0.2 MB. Copyright © 2018 Ishigaki et al. 2018 Ishigaki et al. This content is distributed under the terms of the Creative Commons Attribution 4.0 International license.

10.1128/mSphere.00417-17.1 TEXT S1  Supplemental materials and methods. Download TEXT S1, DOCX file, 0.05 MB. Copyright © 2018 Ishigaki et al. 2018 Ishigaki et al. This content is distributed under the terms of the Creative Commons Attribution 4.0 International license.

### BPI reaction.

All plasmids were transferred to *B. pertussis* strains by triparental conjugation with the *E. coli* helper strain HB101 carrying pRK2013, as described elsewhere (38). For example, *E. coli* DH5α carrying pBPI and HB101 carrying pRK2013 were inoculated into LB with kanamycin. Bp^attP^*ΔrecA*::*gfp* carrying pBBR1MCS5-Int was inoculated into SS broth containing gentamicin. After overnight incubation, the OD values of these bacterial cultures were adjusted to 1.0. Eight hundred microliters of *B. pertussis* and 100 μl of each *E. coli* strain were then mixed. The bacterial mixture was centrifuged, and the pellet was suspended in 10 mM MgSO_4_ and spread on LB plates. After a 6-h incubation at 37°C, the colonies were collected and suspended in SS broth. The appropriately diluted suspension was plated on BG agar plates containing ceftibuten and kanamycin for the selection of recombinants that had integrated pBPI into the chromosome. The integration of DNA fragments into the chromosome was confirmed by PCR with PrimeSTAR GXL DNA polymerase (TaKaRa) and the primers listed in Table S2. Briefly, a fresh colony was suspended in 10 μl of deionized and autoclaved water with a toothpick and heated at 98°C for 3 min. A 1-μl aliquot from the boiled supernatant was used for the PCR template.

### LPS purification and Tricine-SDS–PAGE.

LPS was extracted from *B. bronchiseptica* and *B. pertussis* by using a modified hot aqueous phenol extraction method as previously described (39). *B. bronchiseptica* and *B. pertussis* were grown at 37°C in SS broth for 6 and 12 h, respectively. Twenty milliliters of the bacterial culture was centrifuged, and the resulting pellets were suspended in 1 ml of 10 mM Tris-HCl, pH 8.0, containing 2% SDS. The suspension was mixed with 2 μl of recombinant DNase I (5 U/μl; TaKaRa) and 2 μl of PureLink RNase A (40 mg/ml; Invitrogen) and incubated at 37°C for 1 h. After the addition of 1 μl of proteinase K (20 mg/ml; Sigma), the mixture was then incubated at 37°C overnight. Samples were mixed with an equal volume of 90% phenol preheated to 68°C, and mixtures were incubated at the same temperature for 20 min. Following centrifugation, the aqueous layer was dialyzed against deionized and autoclaved water. The dialyzed solutions were centrifuged at 100,000 × *g* for 3 h, and the precipitate was dissolved in 30 µl SDS sample buffer (62.5 mM Tris-HCl [pH 6.8] containing 6% glycerol, 2% SDS, 12.5 mM dithiothreitol, and 0.01% bromophenol blue). Collected LPS was subjected to Tricine-SDS–PAGE by using a 16% separating gel and 4% stacking gel (40). LPS in the gel was visualized by silver staining with the Sil-Best Stain One kit (Nacalai Tesque) according to the manufacturer’s instructions. Samples on the other gels were transferred to a polyvinylidene difluoride membrane (Millipore) and visualized by immunoblotting with rabbit antiserum against *B. bronchiseptica* or *B. pertussis* at dilution ratios of 1:3,000 and 1:5,000, respectively. For immunoblotting, a goat anti-rabbit IgG–horseradish peroxidase (HRP)-conjugated antibody (1:10,000; Jackson) was used as a secondary antibody. Signals were detected with an ECL Western blotting detection system (GE Healthcare). The rabbit antiserum against *B. bronchiseptica* or *B. pertussis* was prepared according to the standard protocol, by injecting formalin-fixed *B. bronchiseptica* or *B. pertussis* into female Kbl:JW rabbits (Kitayama Labs, Japan).

### Assays for sensitivity of bacteria to rat serum and polymyxin B.

The sensitivities of bacteria to serum and polymyxin B were evaluated by previously reported methods, with slight modifications (22, 41). Normal rat serum was purchased from Japan SLC. Polymyxin B sulfate (Wako) was dissolved in deionized and autoclaved water to obtain a stock solution (1 mg/ml). The bacteria for the assays were grown for 12 h in SS broth to the mid-log phase. Approximately 500 CFU of bacteria were incubated in the presence or absence of 90% serum in Hanks’ balanced salt solution at 37°C for 6 h. For the polymyxin B sensitivity assay, approximately 2 × 10^5^ CFU of bacteria were incubated in phosphate-buffered saline (PBS) with the indicated concentrations of polymyxin B at 37°C for 2 h. After each treatment, bacteria were cultivated on BG agar and CFU were enumerated. Survival rates after the serum treatment were expressed in relation to the input bacterial number (as 100%). Survival rates after the polymyxin B treatment were calculated in relation to the bacterial number when incubated without polymyxin B (as 100%).

### Mouse intranasal infection.

All animal experiments were approved by the Animal Care and Use Committee of the Research Institute for Microbial Diseases, Osaka University, and performed in accordance with the Regulations on Animal Experiments at Osaka University. Bp^attP^*ΔrecA*::*gfp*-pBPI-Gm and Bp^attP^*ΔrecA*::*gfp*-*wbm* strains were grown for 12 h in SS broth to the mid-log phase and combined in approximately equal bacterial numbers. Seven-week-old BALB/c mice were anesthetized with an intraperitoneal injection of midazolam (2.0 mg/kg), medetomidine (0.3 mg/kg), and butorphanol (5.0 mg/kg). Groups of four mice were intranasally inoculated with 5 × 10^6^ CFU of the bacterial mixture, prepared as described above, in 50 μl of PBS. Mice were sacrificed 8 or 14 days postinfection, and the nasal septum, trachea, and lungs were excised, homogenized in PBS, and plated on BG agar containing ceftibuten and kanamycin or gentamicin to assess CFU. The CI for each organ was calculated as follows: CI = [(output CFU of Bp^attP^*ΔrecA*::*gfp*-*wbm*)/(output CFU of Bp^attP^*ΔrecA*::*gfp*-pBPI-Gm)]/[(input CFU of Bp^attP^*ΔrecA*::*gfp*-*wbm*)/(input CFU of Bp^attP^*ΔrecA*::*gfp*-pBPI-Gm)]. A CI of >1 indicated that Bp^attP^*ΔrecA*::*gfp*-*wbm* outcompeted Bp^attP^*ΔrecA*::*gfp*-pBPI-Gm.
