# Supplementary figures and images for: Ectopic Expression of O Antigen in Bordetella pertussis by a Novel Genomic Integration System
Source: mSphere. 2018 Jan 24;3(1):e00417-17. doi: 10.1128/mSphere.00417-17 (PMC5784241; doi:10.1128/mSphere.00417-17)

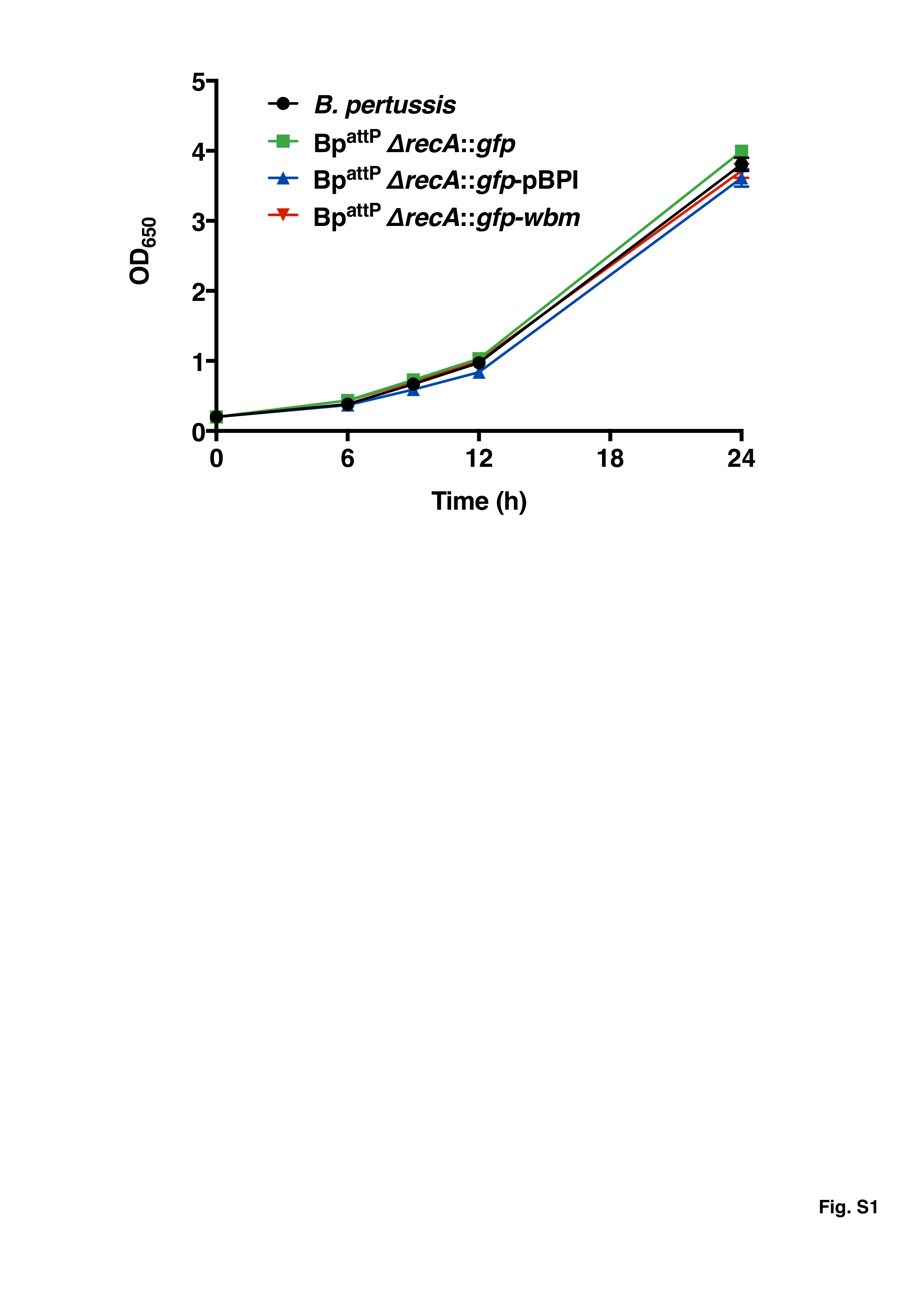

Supplement: FIG S1 [file sph001182454sf1.tif]

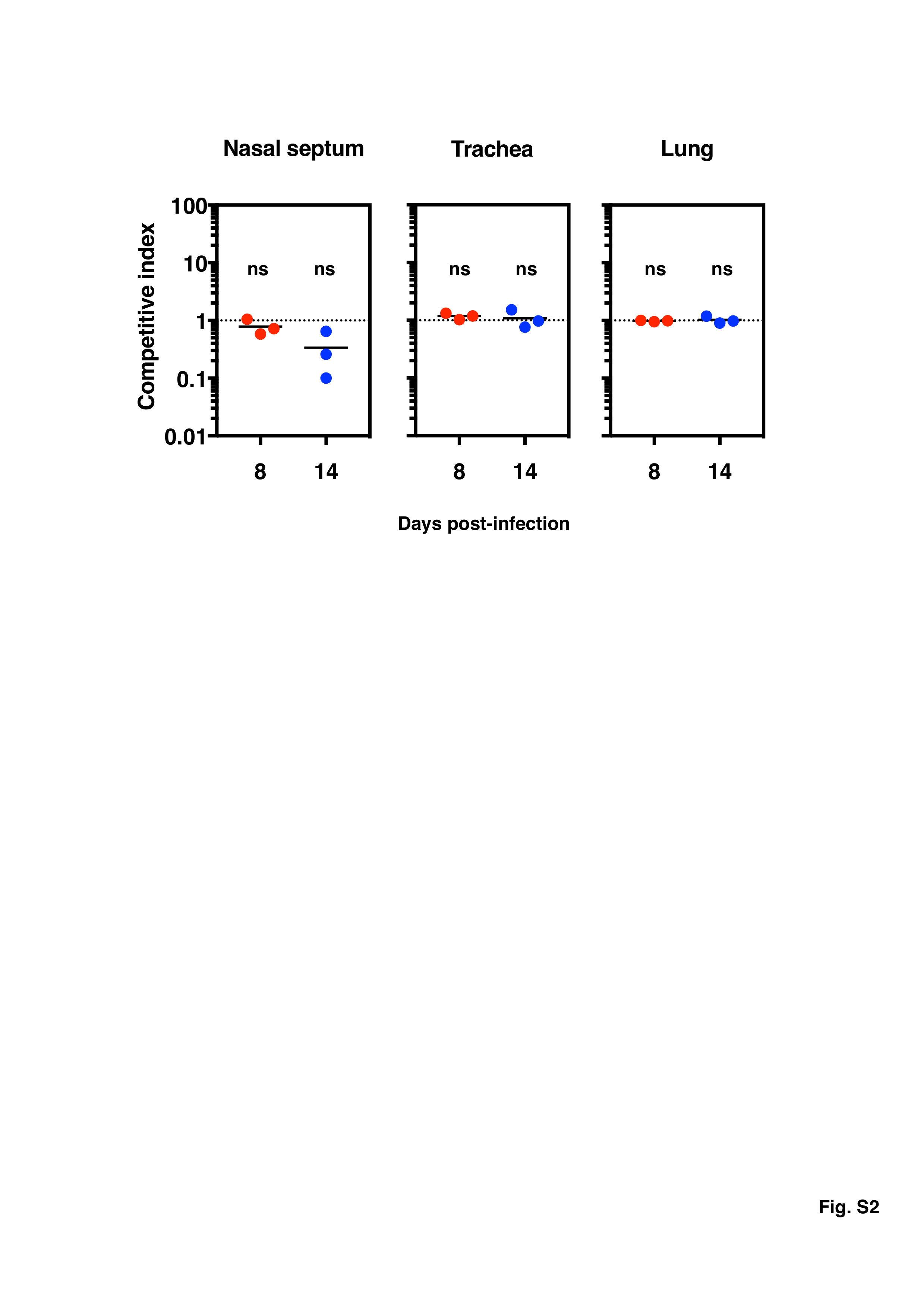

Supplement: FIG S2 [file sph001182454sf2.tif]
