# Supplementary material for: Ectopic Expression of O Antigen in Bordetella pertussis by a Novel Genomic Integration System
Source: mSphere. 2018 Jan 24;3(1):e00417-17. doi: 10.1128/mSphere.00417-17 (PMC5784241; doi:10.1128/mSphere.00417-17)
Supplement: TABLE S1 [file sph001182454st1.docx]

**Supplemental table**

Table S1. Strains and plasmids used in this study

| Strain or plasmid | Description | Source or reference |
| --- | --- | --- |
| ***B. bronchiseptica*** |  |  |
| RB50 | Wild-type | (1) |
|  |  |  |
| ***B. pertussis*** |  |  |
| Tohama I | Wild-type | (2) |
| Bp^attP^ | Tohama I containing an *attP* site in the intergenic region between BP3747 and BP3748 | This study |
| Bp^attP^-pBPI | Bp^attP^ derivative carrying pBPI in the chromosome |  |
| Bp^attP^*ΔrecA*::*gfp* | Bp^attP^ *recA* deletion mutant | This study |
| Bp^attP^*ΔrecA*::*gfp*-pBPI | Bp^attP^*ΔrecA*::*gfp* derivative carrying pBPI in the chromosome | This study |
| Bp^attP^*ΔrecA*::*gfp*-L1 | Bp^attP^*ΔrecA*::*gfp* derivative carrying pBPI-L1 in the chromosome | This study |
| Bp^attP^*ΔrecA*::*gfp-wbm* | Bp^attP^*ΔrecA*::*gfp* derivative carrying pBPI-*wbm* in the chromosome | This study |
| Bp^attP^*ΔrecA*::*gfp*-pBPI-Gm | Bp^attP^*ΔrecA*::*gfp* derivative carrying pBPI-Gm in the chromosome | This study |
|  |  |  |
| ***E. coli*** |  |  |
| HB101 | K-12 cloning strain | Laboratory collection |
| DH5α | K-12 cloning strain | Laboratory collection |
| DH5α λpir | K-12 cloning strain for the plasmid with an R6K origin | Laboratory collection |
|  |  |  |
| **Plasmids** |  |  |
| pRK2013 | Km^r^, RK2-derivative with ColE1 replicon containing *tra*, the helper plasmid for conjugative transfer | (3) |
| pBlueScript KS(+) | Amp^r^, basic cloning vector | Stratagene |
| pJTI R4 DEST | Carrying the R4 *attB* site | Invitrogen |
| pJTI/Neo | Carrying the R4 *attP* site | Invitrogen |
| pJTI PhiC31Int | Carrying PhiC31 integrase | Invitrogen |
| pBBR1-P*tac*-GFP | pBBR1MCS5 carrying the *tac* promoter, *gfp*, and *trpA* terminator | (5) |
| pBBR1MCS2 | Km^r^, broad-host-range cloning vector | (4) |
| pBBR1MCS2-Int | pBBR1MCS2, carrying the *tac* promoter, *integrase*, and *trpA* terminator | This study |
| pBBR1MCS5 | Gm^r^, broad-host-range cloning vector | (4) |
| pBBR1MCS5-Int | pBBR1MCS5, carrying the *tac* promoter, *integrase*, and *trpA* terminator | This study |
| pABB-CRS2-Gm | Gm^r^, R6K-derived suicide vector | Laboratory collection |
| pABB-BP3747-3748 | pABB-CRS2-Gm derivative containing the region between BP3747 and BP3748 | This study |
| pABB-attP | pABB-BP3747-3748 derivative for the introduction of *attP* | This study |
| pABB-*ΔrecA*::*gfp* | pABB-CRS2-Gm derivative for the deletion of *recA* | This study |
| pMIN136T | pBP136 derivative containing the cloning site, oriT from RK2 | (5) |
| pBeloBAC11 | Cm^r^, F plasmid-based low copy vector | NEB |
| pBeloBAC11-Km | Km^r^, pBeloBAC11 derivative | This study |
| pBPI | pBeloBAC11-Km derivative containing *attB* and oriT  (Containing two *Hind*III sites) | This study |
| pBPIori | pBeloBAC11-Km derivative containing *attB*, oriT, and the ColE1 ori (Containing three *Hind*III sites) | This study |
| pBPI-L1 | pBPI containing Bb gDNA | This study |
| pBPI-*Hin*dIII-1 | pBPI derivative containing one *Hin*dIII site | This study |
| pBPI-*Hin*dIII-2 | pBPIori derivative containing two *Hin*dIII sites | This study |
| pBPI-*wbm* | pBPI containing the *wbm* locus | This study |
| pBPI-Gm | Gm^r^, pBPI derivative | This study |
| pBPIori-Gm | Gm^r^, pBPIori derivative | This study |

1. **Cotter PA**, **Miller JF**. 1994. BvgAS-mediated signal transduction: analysis of phase-locked regulatory mutants of *Bordetella bronchiseptica* in a rabbit model. Infect Immun **62**:3381–3390.

2. **Kasuga T, Nakase Y, Ukishima K, Takatsu K.** 1954. Studies on Haemophilus pertussis. V. Relation between the phase of bacilli and the progress of the whooping-cough. Kitasato Arch Exp Med 27:57–62.

3. **Figurski DH**, **Helinski DR**. 1979. Replication of an origin-containing derivative of plasmid RK2 dependent on a plasmid function provided in *trans*. Proc Natl Acad Sci U S A **76**:1648–1652.

4. **Kovach ME**, **Elzer PH**, **Hill DS**, **Robertson GT**, **Farris MA**, **Roop RM**, **Peterson KM**. 1995. Four new derivatives of the broad-host-range cloning vector pBBR1MCS, carrying different antibiotic-resistance cassettes. Gene **166**:175–176.

5. **Nishikawa S**, **Shinzawa N**, **Nakamura K**, **Ishigaki K**, **Abe H**, **Horiguchi Y**. 2016. The *bvg*-repressed gene *brtA*, encoding biofilm-associated surface adhesin, is expressed during host infection by *Bordetella bronchiseptica*. Microbiol Immunol **60**:93–105.
