# Supplementary material for: Ectopic Expression of O Antigen in Bordetella pertussis by a Novel Genomic Integration System
Source: mSphere. 2018 Jan 24;3(1):e00417-17. doi: 10.1128/mSphere.00417-17 (PMC5784241; doi:10.1128/mSphere.00417-17)
Supplement: TABLE S2 [file sph001182454st2.docx]

**Supplemental table**

Table S2. Primers used in this study.

*Underlined sequences are reverse-complement sequences for the In-Fusion cloning.
