# Supplementary material for: Ectopic Expression of O Antigen in Bordetella pertussis by a Novel Genomic Integration System
Source: mSphere. 2018 Jan 24;3(1):e00417-17. doi: 10.1128/mSphere.00417-17 (PMC5784241; doi:10.1128/mSphere.00417-17)
Supplement: TEXT S1 [file sph001182454s1.docx]

**Supplemental materials and methods**

**Construction of plasmids and mutant strains.**

Mutant strains of *B. pertussis* were constructed as described previously (1). The plasmids and primers used in this study are listed in Tables S1 and S2, respectively. Unless otherwise stated, PCR was performed with KOD FX Neo (TOYOBO). For example, Bp^attP^ was constructed as follows. An approximately 2-kbp fragment including the 3’ regions of oppositely-directed BP3747 and BP3748 was amplified with the primer set of pABB-3747-3748-F and pABB-3747-3748-R, and *B. pertussis* Tohama I genomic DNA (gDNA) as a template. Inverse PCR was performed with the primers pABB-CRS2-Gm-inverse-S and pABB-CRS2-Gm-inverse-AS, and pABB-CRS2-Gm as a template. These PCR products were ligated by the In-Fusion HD cloning kit (Clontech Laboratories) according to the manufacturer’s instructions. The resultant plasmid was designated as pABB-BP3747-3748, which was subsequently used as a template for inverse PCR with the primer set of BP3747-3748 insertion-F and BP3747-3748 insertion-R. *attP* was amplified with the primer set of attP-F and attP-R, and pJTI/Neo (Invitrogen) as a template. The *attP* fragment and linear pABB-BP3747-3748 were ligated by the In-Fusion HD cloning kit. The resultant plasmid (pABB-attP) was introduced into *E. coli* DH5α λpir, and then transferred to *B. pertussis* Tohama I by triparental conjugation with a helper strain, HB101/pRK2013. As a result, *attP* was introduced into the intergenic region between BP3747 and BP3748 by two-step homologous recombination. Ceftibuten was used for the counter-selection for *Bordetella*.

Bp^attP^*ΔrecA*::*gfp* was constructed as follows. An approximately 3.1-kbp region containing *recA* was amplified by PCR with the primer set of recA-F and recA-R, and *B. pertussis* Tohama I gDNA as a template. Inverse PCR was performed with the primers pABB-CRS2-Gm-inverse-S and pABB-CRS2-Gm-inverse-AS, and pABB-CRS2-Gm as a template. These PCR products were ligated using the In-Fusion HD cloning Kit and then used as a template for inverse PCR with the primer set of recA-inverse-F and recA-inverse-R. An approximately 1-kbp region containing *gfp* was amplified by PCR with the primer set of Ptac-GFP-F and TtrpA-R, and pBBR1-P*tac*-GFP as a template. The GFP fragment and linear inverse PCR product were ligated using the In-Fusion HD cloning Kit. The resultant plasmid designated as pABB-*ΔrecA*::*gfp* was introduced into *E. coli* DH5α λpir, and then transferred to *B. pertussis* Tohama I by triparental conjugation. *recA* was replaced with *gfp* by two-step homologous recombination. Ceftibuten was used for the counter-selection for *Bordetella*.

pBBR1MCS5-Int was constructed as follows. An approximately 1.4-kbp region containing *int* was amplified by PCR with the primer set of Int-F and Int-R, and pJTI PhiC31Int as a template. Inverse PCR was performed with the primers Inverse-Int-F and Inverse-Int-R, and pBBR1-P*tac*-GFP as a template. These PCR products were ligated using the In-Fusion HD cloning kit.

pBBR1MCS2-Int was constructed as follows. pBBR1MCS5-Int was digested with *Xho*I and *Spe*I to obtain the fragment containing *int*. pBBR1MCS2 was digested with *Xho*I and *Spe*I. These fragments were ligated with T4 DNA Ligase (Promega).

pBeloBAC11-Km was constructed as follows. An approximately 1.2-kbp region containing the kanamycin (Km)-resistant gene was amplified by PCR with the primer set of KmR-F and KmR-R, and pMIN136T (1) as a template. Inverse PCR was performed with the primers KmR-replacement-F and KmR-replacement-R, and pBeloBAC11 as a template. These PCR products were ligated using the In-Fusion HD cloning kit.

pBPI was constructed as follows. An approximately 360-bp region containing *attB* was amplified by PCR with the primer set of attB-F and attB-R, with pJTI R4 DEST as a template. pBeloBAC11-Km was digested with *Hpa*I. These fragments were ligated by the In-Fusion HD cloning kit. The resultant plasmid was digested with *Bst*PI. An approximately 400-bp region containing the oriT sequence was amplified by PCR with the primer set of oriT-F and oriT-R, and pMIN136T as a template. The amplified fragment containing oriT was ligated with the *Bst*PI-digested vector by the In-Fusion HD cloning kit. The resultant pBPI was transferred to Bp^attP^*ΔrecA*::*gfp* by triparental conjugation. The clones that harbor pBPI integrated into the chromosome were selected by cultures on BG agar containing ceftibuten and kanamycin.

pBPIori was constructed as follows. An approximately 1.6-kbp region containing the ColE1-derived origin of replication was amplified by PCR with the primer set of pBSKS-ori-F and pBSKS-ori-R, and pBlueScript KS(+) as a template. The PCR product was ligated with *Bam*HI-digested pBPI by the In-Fusion HD cloning kit.

pBPI and pBPIori have two and three *Hin*dIII sites, respectively. In the cloning of the *wbm* locus, we eliminated the *Hin*dIII site from pBPI and pBPIori as follows. Inverse PCR was performed with the primers HindIII-remove-F and HindIII-remove-R, and pBPI as a template. The PCR product was self-ligated by the In-Fusion HD cloning kit. The resultant vector was named pBPI-*Hin*dIII-1. pBPI-*Hin*dIII-2 was constructed with pBPIori in a similar manner.

pBPI-*wbm* was constructed as follows. PCR was performed with PrimeSTAR GXL DNA Polymerase (TaKaRa). An approximately 10-kbp region including *wbmA* and *wbmH* was amplified by PCR with the primer set of O-antigen-1 and O-antigen-2, and *B. bronchiseptica* RB50 gDNA as a template. The PCR product was ligated with *Hin*dIII/*Bam*HI-digested pBPI-*Hin*dIII-2 by the In-Fusion HD cloning kit. The resultant plasmid containing a single *Hin*dIII site was digested with *Hin*dIII and ligated with an approximately 10-kbp region including *wbmI* and *wbmN*, which had been amplified by PCR with the primer set of O-antigen-3 and O-antigen-4, and *B. bronchiseptica* RB50 gDNA as a template. The resultant vector was digested with *Hin*dIII, and ligated with a fragment of an approximately 11-kbp region between *wbmO* and *BB0121* that had been amplified with the primer set of O-antigen-5 and O-antigen-6 by the In-Fusion HD cloning kit. pBPI-*wbm* was transferred to Bp^attP^*ΔrecA*::*gfp* by triparental conjugation. The clones that carry pBPI-*wbm* integrated into the chromosome were selected by cultivation on BG agar containing ceftibuten and kanamycin.

In the competitive infection assay, the Km-resistant gene of pBPI-*Hin*dIII-2 was replaced with the gentamicin (Gm)-resistant gene. The Gm-resistant gene was amplified by PCR with the primer set of Gm-replace-F and Gm-replace-R, and pBBR1MCS5 as a template. The PCR product was ligated with *Eco*T22I-digested pBPI-*Hin*dIII-2. The resultant vector was designated as pBPIori-Gm. pBPIori-Gm was digested with *Bam*HI and self-ligated to construct pBPI-Gm. pBPI-Gm was transferred to Bp^attP^*ΔrecA*::*gfp* by triparental conjugation. The clones that carry pBPI-Gm integrated into the chromosome were selected by cultivation on BG agar containing ceftibuten and gentamicin.

**Bacterial growth assay**

Bacteria were grown at 37°C on BG agar for 3 days, suspended in SS broth at an OD_650_ value of 0.2, and incubated at 37°C. OD_650_ values were measured after 6-, 9-, 12-, and 24-h incubations.

**Preparation of large genomic DNA fragments**

Large genomic DNA fragments were prepared as described previously with slight modifications (2, 3). *B. bronchiseptica* was suspended in 10 ml of SS broth at an OD_650_ value of 0.2, and grown at 37°C. After 12-15 h, the bacterial culture was centrifuged at 4,000x*g* for 5 min, and the supernatant was discarded. The pellet was resuspended in 10 mM Tris-HCl, pH 8.0, containing 1M NaCl and 50 mM EDTA to give an OD_650_ value of 20, and mixed with an equal volume of pre-heated 1.6% low-melting point (LMP) agarose (LONZA, SeaPlaque™ Agarose). The mixture was transferred into plug molds (Bio-Rad) and allowed to solidify at 4°C. The solidified plugs were incubated in 10 ml of 10 mM Tris-HCl, pH 8.0, containing 1 M NaCl, 100 mM EDTA, 1 mg/ml lysosome, 0.5% N-lauroyl sarcosine sodium salt (Sigma), 0.2% sodium deoxycholate (Nacalai Tesque), and 2 μg/ml RNaseA (Invitrogen) at 37°C for 2 h. After rinsing with deionized and autoclaved water, the plugs were incubated in 10 ml of 0.425 M EDTA, pH 8.0, containing 1 mg/ml Proteinase K (Sigma) and 1% N-lauroyl sarcosine sodium salt (Sigma), at 50°C overnight. After rinsing with deionized and autoclaved water, the plugs were incubated in 50 ml of 20 mM Tris-HCl, pH 8.0, containing 50 mM EDTA (wash solution) at room temperature for 1 h and subsequently in 20 ml of wash solution containing 1mM phenylmethylsulfonyl fluoride (PMSF, Nacalai Tesque). The plugs were rinsed with wash solution for 1 h twice.

The 16 plugs were equilibrated with 3 ml of 1×Cutsmart buffer solution (NEB) on ice for 1 h in a 5-ml polystyrene tube. Ten microliters of *Bam*HI-HF (20 U/ μl, NEB) was added to the buffer solution and the plugs were allowed to be infiltrated with the enzyme by a further incubation on ice for 1 h. The tubes containing the plugs were incubated at 37°C with gentle shaking for exactly 30 min. The tubes were immediately placed on ice and 30 μl of 0.5 M EDTA, pH 8.0, was added into each tube to stop the reaction.

The pieces of the digested plugs were transferred into a pulsed-field certified agarose gel (Bio-Rad). Pulsed-field gel electrophoresis (PFGE) was performed under the following conditions: buffer temperature = 12°C, volts/cm = 6.0, included angle = 120°, initial switch time = 1.0 sec, final switch time = 40.0 sec, running time = 18 h for the first size selection. After electrophoresis, the piece of the gel containing DNA fragments of approximately 100 kbp to 300 kbp, which were estimated by the lambda ladder PFG marker (NEB), was excised, and one third of the piece was subjected to PFGE again under the following conditions: buffer temperature = 12°C, volts/cm = 6.0, included angle = 120°, initial switch time = 0.5 sec, final switch time = 5.0 sec, running time = 18 h for the second size selection. Gel blocks were excised from the PFGE gel and gDNA was extracted from these blocks to Seamless Cellulose Tubing, Small Size 18 (Wako) by electro-elution with 5 V/cm at 4°C for 2 h. The concentration of gDNA obtained was estimated by comparing the intensity of the DNA band with that of lambda DNA of a known concentration after electrophoresis.

**BAC library construction**

pBPIori was digested with *Bam*HI-HF to remove the ColE1 ori, and ligated with the high-molecular-weight gDNA of *B. bronchiseptica* by TaKaRa DNA Ligation Kit LONG (TaKaRa) according to the manufacturer’s instructions. After a 16-h incubation at 16°C, the ligation mixture was dialyzed against 0.1×TE buffer for 3 h. The vectors carrying the gDNA of *B. bronchiseptica* were introduced into *E. coli* HST08 Premium Electro-Cells (TaKaRa) with CELL PORATER (GIBCO-BRL). The transformants carrying gDNA were selected by kanamycin resistance and alpha complementation. In order to evaluate the approximate sizes of the inserted fragments, randomly selected clones were incubated in 20 ml of LB containing 10 μg/ml of kanamycin, and plasmids were extracted with the FastGene Plasmid Mini Kit (Nippon Genetics). The extracted plasmids were digested with *Bam*HI-HF and subjected to electrophoresis.

**Elucidation of the inserted genomic region of *B. bronchiseptica***

*B. bronchiseptica* gDNA was inserted into the *Bam*HI site of pBPI, which had M13 forward and M13 reverse primer sites at the flanking regions. In order to elucidate the sequences of both ends of the inserted region, extracted plasmids were directly sequenced using M13 forward and M13 reverse primers at the core instrumental facility of the Research Institute of Microbial Diseases, Osaka University. One of the clones containing an insert with a length of approximately 49.6 kbp was picked up and named pBPI-L1.

**Mouse intranasal infection**

All animal experiments were approved by the Animal Care and Use Committee of the Research Institute for Microbial Diseases, Osaka University and performed in accordance with the Regulations on Animal Experiments at Osaka University. Bp^attP^*ΔrecA*::*gfp*-pBPI and Bp^attP^*ΔrecA*::*gfp*-pBPI-Gm were grown for 12 h in SS broth to the mid-log phase and combined in approximately equal bacterial numbers. Seven-week-old Balb/c mice were anesthetized with an intraperitoneal injection of midazolam (2.0 mg/kg), medetomidine (0.3 mg/kg), and butorphanol (5.0 mg/kg). Groups of four mice were intranasally inoculated with 5×10^6^ CFU of the bacterial mixture prepared as described above in 50 μl of PBS. Mice were sacrificed 8 or 14 days post-infection and the nasal septum, trachea, and lungs were excised, homogenized in PBS, and plated on BG agar containing ceftibuten and kanamycin or gentamicin to assess CFU. The competitive index (CI) in each organ was calculated as follows: CI = (the output CFU of Bp^attP^*ΔrecA*::*gfp*-pBPI / the output CFU of Bp^attP^*ΔrecA*::*gfp*-pBPI-Gm) / (the input CFU of Bp^attP^*ΔrecA*::*gfp*-pBPI / the input CFU of Bp^attP^*ΔrecA*::*gfp*-pBPI-Gm). CI >1 indicates that Bp^attP^*ΔrecA*::*gfp*-pBPI outcompeted Bp^attP^*ΔrecA*::*gfp*-pBPI-Gm.

**Supplemental references**

1. **Nishikawa S**, **Shinzawa N**, **Nakamura K**, **Ishigaki K**, **Abe H**, **Horiguchi Y**. 2016. The *bvg*-repressed gene *brtA*, encoding biofilm-associated surface adhesin, is expressed during host infection by *Bordetella bronchiseptica*. Microbiol Immunol **60**:93–105.

2. **Farrar K**, **Donnison IS**. 2007. Construction and screening of BAC libraries made from *Brachypodium* genomic DNA. Nat Protoc **2**:1661–1674.

3. **Osoegawa K**, **Mammoser AG**, **Wu C**, **Frengen E**, **Zeng C**, **Catanese JJ**, **de Jong PJ**. 2001. A bacterial artificial chromosome library for sequencing the complete human genome. Genome Res **11**:483–496.
